# *T*elehealth exercise to *I*mprove *P*hysical function and frailty in patients with multiple myeloma treated with autologous hematopoietic *S*tem cell transplantation (TIPS): protocol of a randomized controlled trial

**DOI:** 10.1186/s13063-022-06848-y

**Published:** 2022-11-03

**Authors:** Kyuwan Lee, Nitya Nathwani, Justin Shamunee, Lanie Lindenfeld, F. Lennie Wong, Amrita Krishnan, Saro Armenian

**Affiliations:** 1 Division of Outcomes Research, Department of Population Sciences, Beckman Research Institute, City of Hope Comprehensive Cancer Center, 1500 E. Duarte Rd., Bldg. 173, Duarte, CA 91010 USA; 2 Division of Multiple Myeloma, Department of Hematology and Hematopoietic Cell Transplantation, City of Hope Comprehensive Cancer Center, Duarte, CA 91010 USA

**Keywords:** Telehealth exercise, Physical function, Frailty, Multiple myeloma

## Abstract

**Background:**

Advances in autologous hematopoietic stem cell transplantation (HSCT) and supportive care have led to marked improvements in survival for patients with multiple myeloma. Despite these improvements, patients with multiple myeloma remain at high risk of physical dysfunction and frailty due to HSCT and its associated exposures. Although traditional supervised exercise programs can improve frailty in cancer patients and survivors, rehabilitation facilities are typically far from a patient’s residence, are offered on fixed days/hours, contain uniform activities for everyone, and carry a higher risk of contact cross-infection due to immunosuppression, which can be barriers to exercise participation. Innovative personalized interventions are needed to overcome the limitations of traditional exercise interventions. The purpose of this study is to determine the efficacy and sustainability of a telehealth exercise intervention on physical function and frailty in patients with multiple myeloma treated with HSCT.

**Methods:**

This randomized controlled trial will assess the efficacy of an 8-week telehealth exercise intervention in 60 patients with multiple myeloma who underwent autologous HSCT (30–180 days post-transplant) and are pre-frail or frail. There will be 30 intervention participants and 30 delayed controls. We will administer remote baseline assessments (week 0), followed by an 8-week telehealth intervention (week 1–8), post assessment (week 9), and an additional follow-up assessment (week 17). Our primary endpoint will be improved physical function, as assessed by the Short Physical Performance Battery test. Our secondary endpoint will be a decrease in frailty characteristics such as gait speed, strength, and fatigue. We will also evaluate the sustainability of improved physical function and frailty at week 17. Participants randomized to the intervention group will perform at least 90 min of exercise per week throughout the 8 weeks.

**Discussion:**

This study will help optimize the delivery of safe, low-cost, and scalable telehealth exercise interventions to improve health outcomes in patients with multiple myeloma, an understudied population at high risk for physical dysfunction and frailty. Our study may provide the foundation for sustainable telehealth exercise interventions to improve physical function and frailty for other hematologic cancer patients (e.g., acute leukemia, lymphoma) as well as any other cancer population of interest.

**Trial registration:**

ClinicalTrials.govNCT05142371. This study was retrospectively registered on December 2nd, 2021, and is currently open to accrual.

**Supplementary Information:**

The online version contains supplementary material available at 10.1186/s13063-022-06848-y.

## Background

Advances in autologous hematopoietic stem cell transplantation (HSCT) and supportive care have led to marked improvements in survival for patients with multiple myeloma [1]. In fact, multiple myeloma is the most common indication for autologous HSCT, with an estimated 67% of patients with multiple myeloma comprising the total HSCT in the USA annually [2]. Despite these improvements, patients with multiple myeloma remain at high risk of physical dysfunction and frailty due to HSCT [3], and its associated exposures [4]. Frailty can be defined as having three or more of the following: clinically underweight, exhaustion, low energy expenditure, slow gait speed, and weak strength [3, 5, 6]. It is estimated that two thirds of patients with multiple myeloma have aging-related health conditions such as frailty [7]. The International Myeloma Working Group reported that frail patients with multiple myeloma have the lowest survival rate after 3 years (57% vs 84%) as well as the highest treatment discontinuation (31.2% vs 16.5%) compared to non-frail patients [8]. Additionally, frailty is associated with a 2.7-fold higher risk of mortality in HSCT survivors when compared with those who are not frail [3], which further emphasizes the need for innovative prevention strategies to mitigate these modifiable complication risks after autologous HSCT.

Exercise interventions for hematological cancer survivors have been implemented to reduce treatment-related side effects and counteract physical inactivity. Several studies have examined the effects of exercise before [9–11], during [12, 13], or after [14–17] HSCT. However, studies have not utilized a fully remote telehealth exercise approach on physical function and frailty in these patients. Moreover, previous studies have not focused on patients at risk for developing frailty to show the benefits of participating in an exercise intervention in those at highest risk for adverse health outcomes. This unspecified selection of participants may have led to an underestimation of exercise effects, which has hindered the development of optimal exercise interventions for these patients.

Exercise is one of the most effective strategies to improve physical function and frailty in patients with chronic health conditions including heart failure [18], stroke [19], and cancer [20]. Unfortunately, exercise intervention studies presently face substantial challenges in implementation due to the COVID-19 pandemic [21], which has resulted in the closures of exercise facilities due to the need to maintain appropriate social distancing [22]. One potential exercise approach that does not require in-person attendance and can be accessed anytime and anywhere is a telehealth exercise intervention. A telehealth exercise intervention is an effective alternative model of home-based rehabilitation that can possibly produce higher compliance rates and similar efficacy when compared with hospital-based programs [23]. Studies in patients with coronary artery disease [24], and heart failure [25] have shown that a telehealth exercise intervention is equivalent to traditional cardiac rehabilitation in achieving functional improvement, managing risk factors (blood pressure, lipid profile, and body mass index), and improving quality of life, without serious adverse events. Despite strong evidence supporting the safety and effectiveness of home-based cardiac rehabilitation [26, 27], to our knowledge, no studies have used fully remote exercise interventions to prevent the risk of physical dysfunction and frailty in patients with multiple myeloma.

Therefore, the purpose of this trial is to evaluate a supervised telehealth exercise intervention (at least 30 min/session × 3 days/week for 8 weeks) that is aimed at improving physical function and frailty in patients with multiple myeloma soon after transplant (30–180 days post-transplant). Our primary outcome is physical function, as assessed by the Short Physical Performance Battery test. Our secondary endpoints are frailty characteristics such as gait speed, strength, and fatigue. We will also evaluate the sustainability of improved physical function and frailty at week 17. We will use an innovative telehealth exercise platform (Moterum Technologies, Salt Lake City, USA) which enables remote exercise interventions (e.g., home-based) that are accessible through digital platforms (e.g., video conferencing on smartphones or tablets) and customizable for users to properly address the individual needs of each participant. We hypothesize that the intervention will improve the Short Physical Performance Battery (SPPB) test and 5-scale frailty index, as objectively ascertained using Bluetooth-enabled biosensors and patient-reported questionnaires, respectively.

## Methods/design

**Fig. 1:**
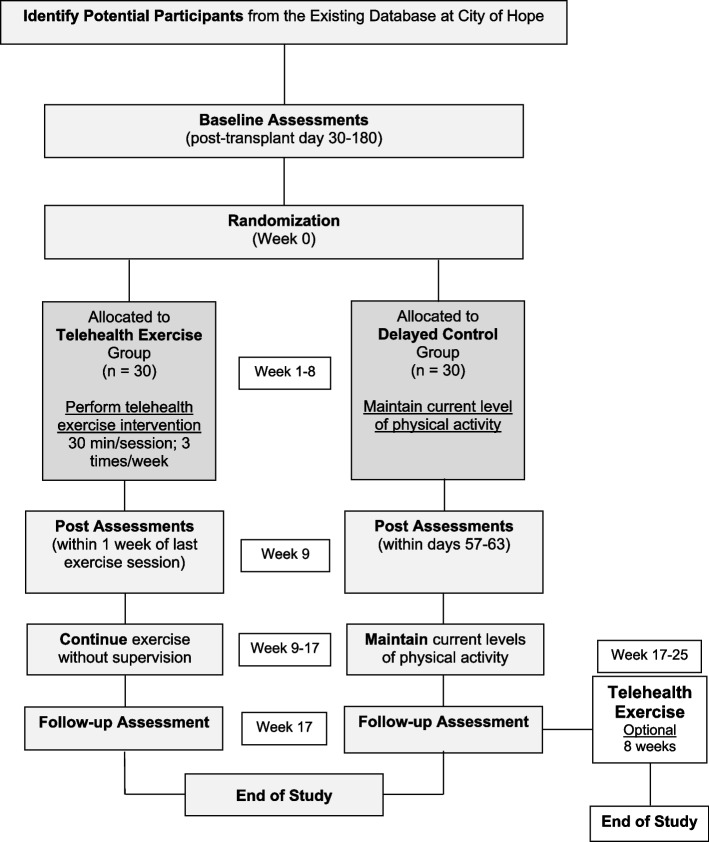
Study flow chart. Participants are randomized into either the telehealth exercise group or the delayed control group for 16 weeks. Participants in the delayed control group are offered the same exercise program after the week 17 assessment

A single-center, investigator-blinded randomized, parallel-group, superiority study was conducted to demonstrate the efficacy of a telehealth exercise intervention on physical function and frailty in patients with multiple myeloma who received HSCT at City of Hope (COH). Figure 1 depicts the flow of the study. We will conduct an 8-week telehealth intervention that will include baseline (week 0) and post-intervention measures (week 9, week 17) on physical function and frailty measures. We will randomize 60 pre-frail or frail patients with multiple myeloma (1:1) to either the 8-week telehealth exercise intervention group (*n*=30) or the delayed control group (*n*=30). All study procedures, including screening and enrollment, will be performed using a telehealth-based approach and will not require participants to be assessed at a clinical center.

### Eligibility

Eligibility requirements include (1) diagnosed with multiple myeloma; (2) ≥18 years of age; (3) self-reported as pre-frail or frail (i.e., Fried criteria: clinically underweight, exhibiting exhaustion, low energy expenditure, slow walking speed, and muscle weakness), with the presence of 2/5 indices classified as pre-frail and ≥3/5 indices classified as frail; (4) within 30 to 180 days post-HSCT prior to registration; (5) physically able and willing to complete all study procedures; and (6) English-speaking. Patients will be excluded if they (1) have clinically significant/active cardiovascular disease (e.g., unstable angina, uncontrolled arrhythmia, etc.); (2) have contraindications to exercise (acute infectious disease or unstable bone lesions); (3) currently recovering from a recent injury or have been physically injured in the past 6 months, in which participation in rigorous exercise may not be appropriate; (4) self-reported ongoing participation of exercise over 60 min per week; and (5) are female patients who are pregnant or planning to become pregnant. As acknowledged in the manuscript, patients who are frail generally have worse outcomes than those who are not, including worse survival and higher treatment discontinuation rates. However, the optimal timing for interventions to reverse frailty is not known. We have elected to include individuals who are pre-frail, as they may represent an optimal phenotype to target for the long-term sustainability of the intervention, prior to the onset of potentially irreversible physical functional decline. We anticipate that the randomized design of the trial will allow for a balanced allocation of pre-frail and frail study participants between the two arms.

### Prescreening/screening

The long-term follow-up after hematopoietic cell transplant program at COH ensures the active and comprehensive follow-up of all patients who have undergone transplant at COH since 1976 — capturing demographics, disease, and vital status, as well as post-transplant complications such as new malignancies, cardiopulmonary disease, and endocrinopathies. Based on our database, we will prescreen all patients diagnosed with multiple myeloma, who will undergo the transplant at COH. After identifying potentially eligible patients, we will inform their primary hematologist at COH to confirm the physician’s clearance before contacting the eligible patients. Each potentially eligible patient will be contacted by phone to determine the patients’ willingness to participate and to identify patient-reported pre-frailty or frailty. If potentially eligible patients do not respond to our initial contact, we will call up to 3 more times, and discontinue our efforts if there is no response. These individuals will be deemed as non-responders

### Recruitment/randomization

If potentially eligible patients are interested in the study, a trained clinical research assistant will confer with the patients in detail about the additional study inclusion/exclusion criteria by phone. Once patients have been deemed eligible for participation, they will complete an informed consent via a web-based consent form (DocuSign™) prior to performing any further testing (see supplementary file). Participants will be randomly assigned to the telehealth exercise intervention group (*n*=30) or delayed control group (*n*=30). Randomization will be performed following physician’s clearance to participate in exercise and baseline outcome assessment using a computer-generated permuted block randomization. An independent biostatistician will be responsible for randomization and study investigators will be blinded to the randomization. Unblinding may need to occur in the case of a medical emergency or a serious medical adverse event study-directed exercise. Unblinding will be done in consultation with the study physician, and the institutional Data Safety Monitoring Board will be notified according to institutional standard operating procedures.

### Remote measures of physical function and frailty

We will mail gait sensors (for hip, right foot, and left foot), a hand dynamometer, measuring tape, and blue tape to each participant’s home. All of this equipment will be used to assist in measuring physical function and frailty outcomes before and after the intervention at each participant’s home, or remote place of choosing, via live video conferencing. Objective data will be recorded by Bluetooth-enabled sensors into the telehealth exercise platform. All participants will complete an initial 30-min technology support session via Zoom video conferencing, during which research staff will help participants set up their sensors and will verify that the sensors operate properly prior to measuring outcome measures. Frailty will be assessed using a 5-scale frailty index: body mass index, fatigue, level of physical activity, gait speed, and muscular strength. Body mass index will be determined by medical record data while fatigue and level of physical activity will be measured by questionnaires. Gait speed will be collected using gait sensors, and hand dynamometer measurements will be used to assess muscular strength. We will assess physical function remotely using the SPPB under the supervision (through videoconferencing) of study staff. SPPB includes three lower extremity measures completed in the following order: timed balance; gait speed; and chair stand. We will assess timed balance under three conditions: (a) side-by-side stand with both feet placed on the ground for 10 s or when the subject steps out of position; (b) semi-tandem stand with the side of the heel of one foot touching the big toe of the other foot for 10 s or when the subject steps out of position; and (c) tandem stand with the heel of one foot touching the big toe of the other foot for 10 s or when the subject steps out of position. We will assess gait speed by instructing participants to measure and mark a 4-meter flat surface distance, across which they will be asked to walk at their “usual” pace. Next, participants will be asked to walk at their “usual” pace to cover the 4-meter distance once more. Time will be recorded using an electronic timer, and the fastest time will be documented. We will assess chair stand under two conditions — participants will (a) perform a single chair stand and (b) be asked to perform five repeated chair stands as quickly as possible; time to completion will be recorded. All participants will complete the remote physical assessments on week 0, week 9, and week 17.

### Telehealth exercise intervention

The 8-week telehealth exercise intervention will begin for the intervention group after participants have completed the baseline assessments and have been randomized. Exercise equipment (resistance bands, resistance loop bands, and exercise mats) will be directly shipped out to the participants from COH. The exercise trainer will assign the appropriate exercises to the participants via the Moterum platform, which will notify each study participant (via email/text) and provide a Zoom link to start a videoconference meeting 15 min prior to each telehealth intervention. We will provide education on safety precautions during the first remote exercise intervention session (i.e., wearing proper shoes, removing clutter, having enough space to move, having a chair near to take breaks when required, and dealing with dizziness, injuries, or falls). The exercise trainer will ensure proper positioning of the camera and also ensure that the workout environment is safe during each exercise session.

A total of 24 video conferences (at least 30 min/session; 3 days/week for 8 weeks) will take place in each participant’s home or remote place of choosing. Of note, the exercise time of 90min/week represents the minimum time for our intervention, and there is no upper limit. Exercise programs will be individualized and prescribed based on the participant’s baseline assessment, physical limitations, and exercise preferences. The exercise prescriptions will be developed and monitored by a clinical exercise physiologist over the course of the intervention. Each exercise session will consist of exercises targeting four essential components: dynamic balance, strength, core stability, and postural control. If a participant cannot complete the exercises as planned, the exercise trainer will provide alternative options and exercise modifications, as needed, to allow the participant to be successful and reach the desired intensity. Allocated interventions will be discontinued based on the participant’s symptoms. For example, if a participant reports any pain related to a prescribed exercise posture or ratings of perceived exertion >8, other exercise postures will be provided. Furthermore, participants will have the option to reschedule or make up an exercise session if they are unable to attend a workout on the date as planned. Exercise adherence for each participant will be captured on the Moterum platform and extracted to assess the feasibility of the prescribed exercise program.

After completing the 8-week telehealth exercise intervention, participants will be encouraged to continue exercising but will no longer be monitored by the exercise trainer for the next 8 weeks until the follow-up physical assessment (week 17). For participants who have successfully completed their remote physical assessments and questionnaires on Week 0 and who are randomized into the delayed control group, they will be asked to continue maintaining their current lifestyle for 17 weeks. On weeks 9 and 17, participants will complete the remote physical assessment and questionnaires again. The study staff will check in with the participants before week 17 and ask them if they would like to participate in the telehealth exercise intervention or not. The exercise program will be offered to the participants as a courtesy and is not mandatory to complete study procedures. All appropriate exercise equipment will be shipped out accordingly to those who would like to participate in the 8-week telehealth exercise program, and outcome measures will not be collected once the program is completed. The study will end after the week 17 assessment for participants in the delayed control group who do not want to participate further with the telehealth exercise option.

### Questionnaires (weeks 0, 9, and 17)

Additional patient-reported outcomes to be measured include instruments that have been found to be reliable, valid, responsive, brief, and easy to administer in cancer patients. All questionnaires will be sent out to the participants electronically via Moterum, and all questionnaires will be self-administered by the patient unless assistance is requested for filling them out. If assistance is preferred, study staff will help over the phone. However, the ability to complete the questionnaires does not influence eligibility criteria.

We will use International Physical Activity Questionnaire, Short Form (IPAQ-SF) to estimate participants’ perception of participating physical activity. That said, we acknowledge that the IPAQ-SF may not detect small changes in levels of physical activity. This questionnaire provides common instruments that can be used to obtain internationally comparable data on health-related physical activity in young and middle-aged adults.

*Quality of life* will be assessed using the Functional Assessment of Cancer Therapy – Multiple Myeloma (FACT-MM) scale developed for the assessment of patient symptoms and Quality of Life in multiple myeloma patients.

*Fatigue* will be assessed using the 13-item FACIT-fatigue scale for the assessment of fatigue/tiredness and its impact on daily activities and functioning in cancer patients.

*Pain* will be assessed by the 15-item Brief Pain Inventory. This self-administered questionnaire measures both the intensity of pain (sensory dimension) and the interference of pain in the patient’s life (reactive dimension). It also asks about pain relief, pain quality, and patient perception of the cause of pain.

*Sleep quality* will be assessed by the Pittsburgh Sleep Quality Index. This is a self-rated questionnaire which assesses sleep quality and disturbances over a 1-month time interval. It looks at subjective sleep quality, sleep latency, sleep duration, habitual sleep efficiency, sleep disturbances, use of sleeping medication, and daytime dysfunction.

### Power calculations

This study is powered to detect a difference in physical function between the two groups after the 8-week intervention. Assuming that a type 1 error = 0.05, data is collected at two time points, there is 20% attrition from baseline to week 9, and there is a correlation of ≥ 0.8 for within-person measurements between time, 60 patients (30 per group) at baseline will achieve 80% power to detect an effect size of at least 0.40 at week 9. A previous study of an 8-week exercise intervention in cancer survivors showed that baseline physical function was 6.7, SD=2.7, with a correlation of 0.9 between baseline and post-intervention [28]. By applying these values to patients with multiple myeloma who underwent HSCT, the present study will be able to show a difference of at least 1 in function score between the treatment groups post-intervention. A 1-unit decrease in the physical function score has been associated with a 12% increase in all-cause mortality in cancer survivors [29], and represents a clinically meaningful difference. The attrition rate in our previous study was 0% [28]. Nevertheless, we conservatively assumed a higher attrition rate of 20% and a lower correlation of 0.70; thus, 60 subjects at baseline (30 telehealth exercise intervention, 30 delayed controls) should provide even greater power since our preliminary study had a higher correlation as well as an attrition of less than 10%.

### Statistical analysis

We will generate descriptive statistics for participants’ demographics, lifestyle behaviors (physical activity, tobacco use), family history of chronic disease, history of other comorbidities (e.g., hypertension, diabetes, dyslipidemia, thyroid disease), and cancer treatment-related exposures. We will assess participation bias by comparing demographics (age, gender, race/ethnicity) and treatment-related exposures among participants and non-participants. Although the number of adverse events is not the major outcome of the study, we will report any adverse events during the study intervention period. We will use a two-sided type I error=0.05. An intention-to-treat analysis will be done by including all randomized participants. We will also conduct another analysis by excluding participants that demonstrate <70% compliance rates within the telehealth exercise sessions completed (17/24). *Hypothesis 1*: The intervention will improve the SPPB test, as objectively measured using Bluetooth-enabled biosensors. We will assess changes in physical function from baseline to week 9 by using the Generalized Estimation Equation (GEE) model with an indicator variable of time, accounting for within-person correlations in measurements. Treatment by time interaction will be included in GEE and its significance tested to assess treatment effects. Body mass index, age, and race will be determined through medical records and included in GEE as a priori covariates for adjustment. Changes in physical function measures from baseline to week 9 will also be examined by a paired *t*-test (within the treatment group). A 2-sided significance level of *p*<0.05 will be used. *Hypothesis 2*: The intervention will improve a 5-scale frailty index, as measured by patient-reported questionnaires. We will assess group differences in changes in the proportion of participants who are of pre-frail or frail from baseline to week 9 using the GEE approach for proportions, accounting for correlation between measurements. As in hypothesis 1, we will include the same covariates for adjustment in the analysis. *Hypothesis 3*: Intervention-related improvements in physical function and frailty will be sustained at week 17. We will examine the long-term effects of the telehealth exercise intervention by fitting GEE models for the outcomes at weeks 9 and 17. The model will include the treatment main effect (to allow for group differences at week 17), time, and the treatment group by time interaction. Contrasts will be used to test if the group difference at week 17 equals that at week 9. As in hypothesis 1, we will include the same covariates for adjustment. Additionally, between-group differences in outcome values at week 17 will be computed, along with 95% confidence intervals, and compared to the differences at week 9. Linear mixed-effects modeling will be used to evaluate the effect of interventions over time on the primary and key secondary outcomes. The models incorporate subject-specific random effects to account for within-subject correlation due to repeated measures. Group averages, as well as subject-specific intercepts and slopes, will be estimated to capture potential variations in the baseline values and slopes among individuals. Linear mixed-effects models accommodate missing data due to dropout, such that all randomized participants can be included. We will compare participants who complete the study with those who do not and identify potential factors that are associated with dropout. An interim analysis is not currently planned for this study.

## Discussion

This study will address current knowledge gaps by including an objective assessment of physical function and frailty before and after a telehealth exercise intervention in patients with multiple myeloma. Physical function and frailty have emerged in recent clinical and epidemiological investigations as intervention targets to improve in hematological cancer survivorship [30–32]. To date, controlled exercise trials in cancer populations have primarily targeted survivors of solid malignancies such as breast [33–35], prostate [36–38], colorectal [39–41], and lung [42–44] cancer. There is a paucity of information on optimal rehabilitation strategies to improve physical function and frailty in patients with multiple myeloma at risk of developing frailty. Moreover, the efficacy and sustainability of fully remote exercise interventions have not been evaluated in this population. The current study presents a unique opportunity to determine the efficacy and sustainability of a telehealth exercise intervention on physical function and frailty in patients with multiple myeloma who have undergone autologous HSCT, which is currently a rapidly growing population at high risk for comorbidities.

Although this study will provide the first evidence of a novel telehealth exercise approach targeting frailty and physical function in patients with multiple myeloma, it is beyond the scope of the current study to elucidate the mechanisms of improving frailty with exercise. Our study has the potential to demonstrate that frailty is maintained and not worsened during the intervention when compared to the delayed control group, which will be a clinically important finding. We recognize that poor retention is a possibility, given the time and effort needed to partake in an intervention study. Therefore, we have accounted for a 20% attrition rate (from randomization to week 17 post-intervention). This takes into consideration patient death, recurrence of cancer, illness, or loss to follow-up. We acknowledge that there may be differential loss to follow-up according to participant attributes and prognosis, which may contribute to participation bias and/or survival bias. To minimize participation bias, we will incorporate many of the strategies (e.g., reminders, incentives) that we have successfully used to maintain excellent participation rates in our exercise intervention studies to date. Attrition will be monitored in real time, allowing us to address obstacles in a timely manner. Differential loss to follow-up due to prognosis, death, and missing data will be addressed using statistical methods of pattern mixture models and multiple imputation. Finally, recruitment of patients with multiple myeloma can be challenging. However, our ongoing successful recruitment strategies in place for the current study, which includes collaborating with the hematology team at COH, appear to be a successful recruitment plan.

This study will provide critical evidence that contributes to the development of comprehensive telehealth exercise programs in HSCT survivors by investigating the effects of an 8-week telehealth exercise intervention in HSCT survivors with the potential of demonstrating improvements and sustainability in physical function. If successful, our approach would be low cost, easily disseminated, and have potential implications for the management and prevention of frailty in HSCT survivors as well as other cancer survivors at risk of developing frailty after cancer treatment.

### Trial status

Protocol version number and date: #211981 (September 29th, 2021), date of first recruitment: October 4, 2021, approximate date to complete recruitment: June 30th, 2023

## Supplementary Information

**Additional file 1.**

## Data Availability

The datasets used and/or analyzed during the current study are available from the corresponding author on reasonable request

## Abbreviations

COH: City of Hope
FACT-MM: Functional Assessment of Cancer Therapy – Multiple Myeloma
HSCT: Hematopoietic stem cell transplantation
SPPB: Short Physical Performance Battery
GEE: Generalized Estimation Equation

## References

[1] Siegel RL, Miller KD, Jemal A (2020). Cancer statistics, 2020. CA Cancer J Clin.

[2] D'Souza A, Fretham C, Lee SJ, Arora M, Brunner J, Chhabra S, Devine S, Eapen M, Hamadani M, Hari P, Pasquini MC, Perez W, Phelan RA, Riches ML, Rizzo JD, Saber W, Shaw BE, Spellman SR, Steinert P, Weisdorf DJ, Horowitz MM (2020). Current use of and trends in hematopoietic cell transplantation in the United States. Biol Blood Marrow Transplant.

[3] Arora M, Sun CL, Ness KK, Teh JB, Wu J, Francisco L, Armenian SH, Schad A, Namdar G, Bosworth A, Kuo L, Weisdorf DJ, Forman SJ, Bhatia S (2016). Physiologic frailty in nonelderly hematopoietic cell transplantation patients: results from the bone marrow transplant survivor study. JAMA Oncol.

[4] Wildes T, Luo SH, Colditz GA, Carson KR. Comorbidities Impact Survival in Multiple Myeloma: Analysis of the Veterans Health Administration National Database. Blood. 2012;120(21):760.

[5] Patel BG, Luo S, Wildes TM, Sanfilippo KM (2020). Frailty in older adults with multiple myeloma: a study of US Veterans. JCO Clin Cancer Inform.

[6] Ness KK, Krull KR, Jones KE, Mulrooney DA, Armstrong GT, Green DM, Chemaitilly W, Smith WA, Wilson CL, Sklar CA, Shelton K, Srivastava DK, Ali S, Robison LL, Hudson MM (2013). Physiologic frailty as a sign of accelerated aging among adult survivors of childhood cancer: a report from the st jude lifetime cohort study. J Clin Oncol.

[7] Engelhardt M, Domm AS, Dold SM, Ihorst G, Reinhardt H, Zober A, Hieke S, Baayen C, Muller SJ, Einsele H, Sonneveld P, Landgren O, Schumacher M, Wasch R (2017). A concise revised Myeloma Comorbidity Index as a valid prognostic instrument in a large cohort of 801 multiple myeloma patients. Haematologica.

[8] Palumbo A, Bringhen S, Mateos MV, Larocca A, Facon T, Kumar SK, Offidani M, McCarthy P, Evangelista A, Lonial S, Zweegman S, Musto P, Terpos E, Belch A, Hajek R, Ludwig H, Stewart AK, Moreau P, Anderson K, Einsele H, Durie BGM, Dimopoulos MA, Landgren O, San Miguel JF, Richardson P, Sonneveld P, Rajkumar SV (2015). Geriatric assessment predicts survival and toxicities in elderly myeloma patients: an International Myeloma Working Group report. Blood.

[9] Jacobsen PB, Le-Rademacher J, Jim H, Syrjala K, Wingard JR, Logan B, Wu J, Majhail NS, Wood W, Rizzo JD, Geller NL, Kitko C, Faber E, Abidi MH, Slater S, Horowitz MM, Lee SJ (2014). Exercise and stress management training prior to hematopoietic cell transplantation: Blood and Marrow Transplant Clinical Trials Network (BMT CTN) 0902. Biol Blood Marrow Transplant.

[10] Keser I, Suyani E, Aki SZ, Sucak AG (2013). The positive impact of regular exercise program on stem cell mobilization prior to autologous stem cell transplantation. Transfus Apher Sci.

[11] Wood WA, Phillips B, Smith-Ryan AE, Wilson D, Deal AM, Bailey C, Meeneghan M, Reeve BB, Basch EM, Bennett AV, Shea TC, Battaglini CL (2016). Personalized home-based interval exercise training may improve cardiorespiratory fitness in cancer patients preparing to undergo hematopoietic cell transplantation. Bone Marrow Transplant.

[12] Jarden M, Baadsgaard MT, Hovgaard DJ, Boesen E, Adamsen L (2009). A randomized trial on the effect of a multimodal intervention on physical capacity, functional performance and quality of life in adult patients undergoing allogeneic SCT. Bone Marrow Transplant.

[13] Mello M, Tanaka C, Dulley FL (2003). Effects of an exercise program on muscle performance in patients undergoing allogeneic bone marrow transplantation. Bone Marrow Transplant.

[14] Knols RH, de Bruin ED, Uebelhart D, Aufdemkampe G, Schanz U, Stenner-Liewen F, Hitz F, Taverna C, Aaronson NK (2011). Effects of an outpatient physical exercise program on hematopoietic stem-cell transplantation recipients: a randomized clinical trial. Bone Marrow Transplant.

[15] Persoon S, Chin AMJM, Buffart LM, Liu RDK, Wijermans P, Koene HR, Minnema MC, Lugtenburg PJ, Marijt EWA, Brug J, Nollet F, Kersten MJ (2017). Randomized controlled trial on the effects of a supervised high intensity exercise program in patients with a hematologic malignancy treated with autologous stem cell transplantation: results from the EXIST study. PLoS One.

[16] Hacker ED, Larson J, Kujath A, Peace D, Rondelli D, Gaston L (2011). Strength training following hematopoietic stem cell transplantation. Cancer Nurs.

[17] Koutoukidis DA, Land J, Hackshaw A, Heinrich M, McCourt O, Beeken RJ, Philpott S, DeSilva D, Rismani A, Rabin N, Popat R, Kyriakou C, Papanikolaou X, Mehta A, Paton B, Fisher A, Yong KL (2020). Fatigue, quality of life and physical fitness following an exercise intervention in multiple myeloma survivors (MASCOT): an exploratory randomised Phase 2 trial utilising a modified Zelen design. Bri J Cancer.

[18] Lee H, Boo S, Yu J, Suh SR, Chun KJ, Kim JH (2017). Physical functioning, physical activity, exercise self-efficacy, and quality of life among individuals with chronic heart failure in Korea: a cross-sectional descriptive study. J Nurs Res.

[19] Moore SA, Hallsworth K, Jakovljevic DG, Blamire AM, He JB, Ford GA, Rochester L, Trenell MI (2015). Effects of community exercise therapy on metabolic, brain, physical, and cognitive function following stroke: a randomized controlled pilot trial. Neurorehabil Neural Repair.

[20] Dittus K, Toth M, Priest J, O'Brien P, Kokinda N, Ades P (2020). Effects of an exercise-based oncology rehabilitation program and age on strength and physical function in cancer survivors. Support Care Cancer.

[21] Schwendinger F, Pocecco E. Counteracting Physical Inactivity during the COVID-19 Pandemic: Evidence-Based Recommendations for Home-Based Exercise. Int J Environ Res Public Health. 2020;17(11):3909. 10.3390/ijerph17113909 PMC7311977 32492778

[22] Phillipou A, Meyer D, Neill E, Tan EJ, Toh WL, Van Rheenen TE, Rossell SL (2020). Eating and exercise behaviors in eating disorders and the general population during the COVID-19 pandemic in Australia: Initial results from the COLLATE project. Int J Eating Disord.

[23] Zwisler AD, Norton RJ, Dean SG, Dalal H, Tang LH, Wingham J, Taylor RS (2016). Home-based cardiac rehabilitation for people with heart failure: A systematic review and meta-analysis. Int J Cardiol.

[24] Bravo-Escobar R, Gonzalez-Represas A, Gomez-Gonzalez AM, Montiel-Trujillo A, Aguilar-Jimenez R, Carrasco-Ruiz R, Salinas-Sanchez P. Effectiveness and safety of a home-based cardiac rehabilitation programme of mixed surveillance in patients with ischemic heart disease at moderate cardiovascular risk: A randomised, controlled clinical trial. Bmc Cardiovascular Disorders. 2017;17(1):66. 10.1186/s12872-017-0499-0 PMC5319164 28219338

[25] Imran HM, Baig M, Erqou S, Taveira TH, Shah NR, Morrison A, et al. Home-Based Cardiac Rehabilitation Alone and Hybrid With Center-Based Cardiac Rehabilitation in Heart Failure: A Systematic Review and Meta-Analysis. J Am Heart Assoc. 2019;8(16):e012779. 10.1161/JAHA.119.012779 PMC6759908 31423874

[26] Bowman A, Denehy L, Benjemaa A, Crowe J, Bruns E, Hall T, et al. Feasibility and safety of the 30-second sit-to-stand test delivered via telehealth: an observational study. PM&R. 2022. 10.1002/pmrj.12783 35138036

[27] Ng CA, McMillan LB, Humbert L, Ebeling PR, Scott D (2021). Feasibility, safety and effectiveness of a pilot 16-week home-based, impact exercise intervention in postmenopausal women with low bone mineral density. Osteoporos Int.

[28] Lee K, Kang I, Mack WJ, Mortimer J, Sattler F, Salem G, Dieli-Conwright CM (2019). Feasibility of high intensity interval training in patients with breast Cancer undergoing anthracycline chemotherapy: a randomized pilot trial. BMC Cancer.

[29] Brown JC, Harhay MO, Harhay MN (2015). Physical function as a prognostic biomarker among cancer survivors. Bri J Cancer.

[30] Armenian SH, Horak D, Scott JM, Mills G, Siyahian A, Teh JB, Douglas PS, Forman SJ, Bhatia S, Jones LW (2017). Cardiovascular Function in Long-Term Hematopoietic Cell Transplantation Survivors. Biol Blood Marrow Transplant.

[31] Mohammed J, Smith SR, Burns L, Basak G, Aljurf M, Savani BN, Schoemans H, Peric Z, Chaudhri NA, Chigbo N, Alfred A, Bakhsh H, Salooja N, Chris Chim A, Hashmi SK (2019). Role of physical therapy before and after hematopoietic stem cell transplantation: white paper report. Biol Blood Marrow Transplant.

[32] Takekiyo T, Dozono K, Mitsuishi T, Murayama Y, Maeda A, Nakano N, Kubota A, Tokunaga M, Takeuchi S, Takatsuka Y, Utsunomiya A (2015). Effect of exercise therapy on muscle mass and physical functioning in patients undergoing allogeneic hematopoietic stem cell transplantation. Support Care Cancer.

[33] Yang A, Sokolof J, Gulati A (2018). The effect of preoperative exercise on upper extremity recovery following breast cancer surgery: a systematic review. Int J Rehabil Res.

[34] Penttinen H, Utriainen M, Kellokumpu-Lehtinen PL, Raitanen J, Sievanen H, Nikander R, Blomqvist C, Huovinen R, Vehmanen L, Saarto T (2019). Effectiveness of a 12-month Exercise Intervention on Physical Activity and Quality of Life of Breast Cancer Survivors; Five-year Results of the BREX-study. In Vivo.

[35] Abdin S, Lavallee JF, Faulkner J, Husted M (2019). A systematic review of the effectiveness of physical activity interventions in adults with breast cancer by physical activity type and mode of participation. Psycho Oncol.

[36] Keilani M, Hasenoehrl T, Baumann L, Ristl R, Schwarz M, Marhold M, Komandj TS, Crevenna R (2017). Effects of resistance exercise in prostate cancer patients: a meta-analysis. Support Care Cancer.

[37] Chaplow ZL, Bowman J, DeScenza VR, Dispennette K, Haynam M, Hohn S, Zhang XC, Focht BC (2020). Effects Of Exercise On Disablement Process Outcomes In Prostate Cancer Patients Undergoing Androgen Deprivation Therapy: An Updated Systematic Review. Med Sci Sports Exerc.

[38] Edmunds K, Tuffaha H, Scuffham P, Galvao DA, Newton RU (2020). The role of exercise in the management of adverse effects of androgen deprivation therapy for prostate cancer: a rapid review. Supportive Care Cancer.

[39] van Rooijen SJ, Engelen MA, Scheede-Bergdahl C, Carli F, Roumen RMH, Slooter GD, Schep G (2018). Systematic review of exercise training in colorectal cancer patients during treatment. Scand J Med Sci Sports.

[40] Lund CM, Dolin TG, Mikkelsen MK, Juhl CB, Vinther A, Nielsen DL (2020). Effect of exercise on physical function and psychological well-being in older patients with colorectal cancer receiving chemotherapy-a systematic review. Clin Colorectal Cancer.

[41] Gao RT, Yu TZ, Liu L, Bi JS, Zhao HY, Tao YJ, Li F, Guo LR (2020). Exercise intervention for post-treatment colorectal cancer survivors: a systematic review and meta-analysis. J Cancer Survivorship.

[42] Zhou WJ, Woo S, Larson JL (2020). Effects of perioperative exercise interventions on lung cancer patients: An overview of systematic reviews. J Clin Nurs.

[43] Rosero ID, Ramirez-Velez R, Lucia A, Martinez-Velilla N, Santos-Lozano A, Valenzuela PL, et al. Systematic Review and Meta-Analysis of Randomized, Controlled Trials on Preoperative Physical Exercise Interventions in Patients with Non-Small-Cell Lung Cancer. Cancers. 2019;11(7):944. 10.3390/cancers11070944 PMC6678369 31284372

[44] Zhou L, Chen QJ, Zhang JY (2021). Effect of exercise on fatigue in patients with lung cancer: a systematic review and meta-analysis of randomized Trials. J Palliat Med.

