# Supplementary material for: Telehealth exercise to Improve Physical function and frailty in patients with multiple myeloma treated with autologous hematopoietic Stem cell transplantation (TIPS): protocol of a randomized controlled trial
Source: Trials. 2022 Nov 3;23:921. doi: 10.1186/s13063-022-06848-y (PMC9633031; doi:10.1186/s13063-022-06848-y)
Supplement: Supplementary file 1 — Additional file 1. [file 13063_2022_6848_MOESM1_ESM.pdf]

ADULT INFORMED CONSENT

COH Protocol #21406

TITLE: Effects of a Telehealth Exercise Intervention to Improve Physical Function and Frailty in Multiple Myeloma Survivors

Protocol Version date: 08/30/2021

PRINCIPAL INVESTIGATOR: Kyuwan Lee, PhD

DAY TIME TELEPHONE NUMBER FROM THE HOURS OF 8:00 AM TO 5:00 PM: (626) 218-6497

EXPERIMENTAL PARTICIPANT'S BILL OF RIGHTS

The rights below are the rights of every person who is asked to be in a research study, also known as an experiment or clinical trial. As a research participant, you have the following rights:

1. To be told what the research study is trying to find out.
2. To be told what will happen to you and whether any of the procedures to be used are different from what would be used in standard practice.
3. To be told about the discomforts, side effects and risks of the things that will happen to you as part of the research study.
4. To be told if you can expect any benefit from participating in the research study.
5. To be told of the other choices you have and how they may be better or worse than being in the research study.
6. To be told what medical treatment is available if any complications arise.
7. To be allowed to ask any questions concerning the research study, both before agreeing to be in the study and during the course of the study.
8. To refuse to participate in the research study or to change your mind about participation after the study is started. To be informed that this decision will not affect your right to receive the care you would receive if you were not in the study.
9. To receive a copy of the signed and dated research study consent form.
10. To be free of pressure when considering whether you wish to agree to be in the research study.

NOT FOR CLINICAL USE

ADULT INFORMED CONSENT

COH Protocol #21406

TITLE: Effects of a Telehealth Exercise Intervention to Improve Physical Function and Frailty in Multiple Myeloma Survivors

PRINCIPAL INVESTIGATOR: Kyuwan Lee, PhD

KEY INFORMATION

You are invited to participate in a research study. The purpose of this research study is to determine if a telehealth exercise program is appropriate for multiple myeloma survivors who had a bone marrow transplant (BMT). This study will also look at how the program affects the health and well-being of those who participate. Telehealth exercise is a service provided online with a care provider. The purpose of the service is to help you perform physical exercises at home. The information we learn from this research study will be valuable in designing future telehealth exercise programs for cancer-related side effects.

Participants in this study will be involved in a home-based exercise program, 3 times per week, for 8 weeks. There will be a baseline assessment before the 1st week along with a follow-up assessment on the 9th week. An additional follow-up physical assessment on the 17th week will take place as well.

A very rare, but major risk associated with the study includes potential discomfort with your breathing during exercise and muscle soreness after the exercise session.

You do not have to be in this research study. Participation is completely optional. If you are interested in learning more about this study, please continue to read below.

INTRODUCTION

You are invited to participate in a clinical trial, which is a type of research study, because you had a BMT and are diagnosed with multiple myeloma. This research study is looking at an exercise program for BMT survivors diagnosed with multiple myeloma. The exercise program uses a telehealth platform (delivered by smart phones, tablets, or computers) to view pre-recorded exercise videos on coordination, posture, stretching, balance, and resistance/aerobic (cardio) training. This study will see if participants are willing to join and are able to successfully complete the 8-week telehealth exercise program. The study will also see whether the program will improve participant's physical function and strength or not. The information we learn by doing this research study will provide information on the best way to conduct a larger study using telehealth exercise programs on health outcomes in cancer survivors.

It is expected that about 60 people will take part in this research study.

NOT FOR CLINICAL USE

We kindly encourage you to take some time to think this over, to discuss it with other people and your primary doctor, and to ask questions now and at any time in the future. Please take as much time as you need to read the consent form. If you find any of the language difficult to understand, please ask questions. If you decide to participate, you will be asked to sign this form. We will give you a copy so that you can refer to it while you are involved in this research study. If you choose not to participate in this research study, the research doctors will discuss other treatment options with you and/or refer you back to your primary doctor.

A. WHY IS THIS RESEARCH STUDY BEING DONE?

This research study is a randomized controlled trial, which aims to examine the effects of the study intervention. In this research study, we are focusing on specific physical health disabilities experienced after BMT called frailty. Frailty includes being underweight, slow walking speed, exhaustion, low physical activity, and weakness. With the use of telehealth exercise, BMT survivors, like yourself, will be able to participate in exercise programs at home.

B. WHAT IS INVOVLED IN THE STUDY?

If you decide to take part, this is what will happen:

Because no one knows which of the study options is best, you will be “randomized” into one of the study groups known as “Arm A” or “Arm B” (see below). Randomization means that you are put into a group by chance. It is like pulling a number out of a hat. Neither you nor the research team will choose what group you will be in, and neither you nor the research team will know what group you are in until only after you have completed the initial assessment. You will have an equal chance of being placed in any of the following groups (explained below).

The initial assessment is the first time you will engage in physical tests and complete questionnaires. The same tests and questionnaires will be done at week 9. Whether you are assigned to Arm A or Arm B, you are asked to participate in these assessments with an exercise trainer who will assist you via a Zoom session while you are at home or a place of your choosing. Zoom is a videoconferencing application. All participants will complete the assessments and questionnaires one more time for a 17-week follow-up.

- **Arm A:** Telehealth exercise group: Participants in this group will perform the initial assessment, the 8-week telehealth exercise intervention, the assessment at week 9, and then will be followed-up 8 weeks later for the final assessment on week 17.

- **Arm B**: Delayed controlled group: Participants in this group will perform the initial assessment, the assessment at 9 weeks, and the assessment at 17 weeks. During the 8-week intervention periods, they will only be asked to continue maintaining their current activities of daily living and will not participate in any exercise program. The 8-week

NOT FOR CLINICAL USE

telehealth exercise intervention will be offered to participants after the final assessment at week 17, if wanted. Participants do not need to participate in the 8-week exercise program if they do not want to.

<u>Before the research starts (screening)</u>:
After signing this consent form, the study staff will make sure you are eligible to participate in this study by asking a few questions about your current health status.

If you are eligible to participate in the research study, you will be enrolled in the study. If you do not meet the eligibility criteria, you will not be able to participate in this research study.

NOT FOR CLINICAL USE

Study Procedures:

If you are eligible to participate in this research study, the following procedures will occur. A calendar summarizing the timing of these tests and procedures is also provided below.

Study Calendar for Participants:

Remote Procedures	Telehealth Exercise Group				Delayed Control Group			
	Week 0 (± 1 Week)	Week 1 (± 1 Week)	Week 9	Week 17	Week 0 (± 1 Week)	Week 1	Week 9	Week 17
Screening	X				X			
Informed Consent	X				X			
Outcome Measures	X		X	X	X		X	X
Questionnaires	X		X	X	X		X	X
Randomization (after Week 0 Outcome Measures/Questionnaires)	X				X			
8-Week Telehealth Exercise		X						*X
8-Week Normal Activities of Daily Living						X		

*8-Week Telehealth Exercise will be optional for the Delayed Control Group.

Procedures for Arms A and B

For Arm A, the study procedures will be performed on three separate occasions: 1) before the 8 weeks of the telehealth exercise program, 2) after the 8 weeks of the telehealth exercise program, and 3) follow-up after 8 weeks have passed from the 2nd assessment.

For Arm B, the study procedures will be performed on three separate occasions: 1) before the 8 weeks of normal activities of daily living, 2) after the 8 weeks of normal activities of daily living, and 3) follow-up after 8 weeks have passed from the 2nd assessment.

Questionnaires: All participants will be asked to complete 6 questionnaires at week 0, week 9, and week 17. Each questionnaire will take approximately 10-20 minutes to complete. The questionnaires cover a variety of topics such as information about your life and medical history, health-related physical activity, quality of life, level of fatigue, level of pain, and quality of sleep.

At-home Physical Assessments: You will have a support session via a Zoom call with a certified personal trainer. During this 30-min session, the trainer will help you set up your sensors (mailed to you in advance) and will verify that the sensors operate properly prior to measuring your at-home

NOT FOR CLINICAL USE

physical assessments. The sensors are small devices that can easily be attached to your waistband and shoes to see how fast you walk. All sensors will be worn throughout the physical assessments and exercise sessions. They can be worn at home outside from these study procedures if you would like to document your steps throughout the day. If you plan to do so, inform the exercise trainer for further information. You will be coached via live video conferencing to complete the following assessments at week 0, week 9, and at week 17:

- **Hand Grip Strength Test** – You will squeeze a device, known as a hand-held dynamometer, as hard as you can in your left hand and then in your right hand. It will be mailed to your home for you to use. The hand-held dynamometer is a device that measures how much force is pushed against it. Through this method, we can measure your strength.

- **Timed Balance Test** – You will stand in 3 different positions (stances) for 10 seconds each. The first stance is called a narrow stance, and it is where your feet will be positioned side by side and touching with both toes pointing forward. The second stance is called a semi tandem stance, and it is where one foot (either left or right foot) is in front of the other. The heel of the front foot is touching the inner side of the big toe of the back foot. The third stance is called a tandem stance. It is like the semi tandem stance except the feet are positioned where the heel of the front foot (either left or right foot) is in front of the big toe of the back foot. You can lose your balance during this test. You should do the tests near a wall or an object that can help you to keep your balance if needed.

- **Gait Speed Test** – You will be asked to walk at your normal pace to cover 4 meters, or about 13 feet. You will do this 2 times, and the exercise trainer will record the time it takes you to complete the walk for each time. We will ask that you are able to clear the floor of objects which may get in your way. A measuring tape and blue tape will be mailed for you to use to measure and mark off the 4-meter (about 13 feet) distance.

- **Chair Stand Test** – You will use a regular chair for this test, and the test is made up of two parts. For the first part, you will remain seated on the chair and then stand up and sit back down without using your arms for assistance. For the second part, you will remain seated on the chair and then stand up as fast as you can and sit back down as fast as you can in a safe manner. Perform this movement for a total of 5 times. We will record the time it takes you to stand up and sit down from the chair 5 times successfully without using your arms for assistance.

Telehealth Exercise Program: All exercise sessions will be accessed on the telehealth platform. The platform automatically notifies you (via email/text) 15 minutes before your scheduled start time and provides an online link to start the videoconference exercise session with the exercise trainer. In addition to exercise guidance, the trainer will provide education on safety precautions during the

NOT FOR CLINICAL USE

home-based exercise session (including setting up equipment, wearing proper shoes and clothing, having enough space to move, having a chair near to take breaks when required, instructions in case of dizziness, injuries, or falls). You will be coached via live video conferencing to complete 15 different exercises per week (five exercise per day) during the 8-week intervention. If you cannot complete the exercises as planned, the exercise trainer will provide other options. There will be a total of 24 video conferencing calls (30 minutes per session; 3 days/week for 8 weeks) which will take place at home. Each exercise session per week is described below:

- **Day 1: Upper Body and Abs –** You will use resistance bands to perform strength exercises for the upper body such as a bicep curl and ab exercises such as a vertical plank.
- **Day 2: Lower Body and Abs –** You will use resistance loop bands to perform strength exercises for the lower body such as a squat and ab exercises such as a vertical plank.
- **Day 3: Total Body –** You will use resistance bands to perform strength exercises for the upper body such as a bicep curl and lower body exercises such as a squat.

Arm A will perform the telehealth exercise program during the 8 weeks of intervention. Arm B will not be given any exercise intervention during the 8 weeks but will have the option to perform the telehealth exercise program after the final assessment (on week 17) is completed. If you are in Arm B and choose to participate in the telehealth exercise program, all exercise sessions must be scheduled to fall within 8 weeks following the week 17 assessment date.

C. <u>HOW LONG WILL I BE IN THIS RESEARCH STUDY?</u>

You will be in this research study for about 17-25 weeks.

D. <u>WHAT ARE THE POSSIBLE RISKS AND DISCOMFORTS?</u>

The risks of this study are as follows:

<u>Risks Associated with Resistance Training Exercise</u>:

Participation in this study requires exercise. Exercise has inherent physical risks associated with it, such as a physical injury or falling. The exercise activities should be done at your own pace and physical ability to minimize potential risks of physical injury. If you have any questions regarding the type of exercise you are performing and any specific risks associated with the exercises, please consult the study doctor.

<u>Risks associated with Breach of Confidentiality:</u>

You will be assigned to a study participation number and your health-related information will be confidentially used based on the study participation number.

NOT FOR CLINICAL USE

Incidental Findings:

It is possible that research procedures of this study may find a medical problem unrelated to the purpose of this study which you did not know about before. If during the research procedures we learn information that may be important for you to know about, such as the possibility of a previously unknown medical condition, we will tell you. You may authorize the release and communication of the findings to your personal doctor. These findings may require additional testing or treatment. You will be responsible for the cost of any additional tests or related treatment.

Reproductive Risks:

We do not know whether this exercise program might hurt an unborn child. While participating in this research study, you should not become pregnant. We can provide counseling about preventing pregnancy for female study participants. Let your doctor know immediately if you become pregnant.

If you become pregnant, you cannot take part in this study. If you are sexually active and capable of bearing a child, both you and your partner must agree to use a medically effective form of birth control while you are on this study. The exercises may involve risks to you (or to the embryo or fetus, if you become pregnant), which is currently unforeseeable.

E. WILL YOU RECEIVE NEW INFORMATION ABOUT THIS STUDY?

During the research study, you will be notified of newly discovered side effects or significant findings, which may affect your health or willingness to participate. You may be asked to sign a new consent form that shows that you have been informed of new information relating to this research study.

F. HOW WILL YOUR INFORMATION BE KEPT CONFIDENTIAL?

Any information learned from this study in which you might be identified will be confidential and disclosed only with your permission. Every effort will be made to keep any information collected about you confidential. However, it is impossible to guarantee that information about you will not be mistakenly released. If, despite our best efforts, identifying information about you is released, it could negatively impact you or your family members. This risk is small.

There are organizations that may inspect your records. These organizations are required to make sure your information is kept private, unless required by law to provide information. Some of these organizations are:

- The Institutional Review Board, also called IRB, is a group of people who review the research with the goal of protecting the people who take part in the study.
- Moterum Technologies, Inc.
- Regulatory agencies such as the Office for Human Research Protections (OHRP), the Food and Drug Administration (FDA), and the National Cancer Institute (NCI) in the U.S., and similar ones if other countries are involved in the study as required by law.

While using the telehealth platform, Moterum, the application may collect data about you, including your name and contact information (used to set up your account). Answers from questionnaires and

NOT FOR CLINICAL USE

measurements of your walking pattern gathered while you are using the sensors will also be stored by Moterum. The data will be collected and transmitted to the researchers and may also be transmitted to people outside of the research study. A complete description of this data collection and sharing is found in the Terms of Use. The Terms of Use provides instructions on how to request deletion of your personal data if you decide to do that in the future. Moterum will also mail you some of the devices you will need for this study. By participating, you are agreeing to let City of Hope provide Moterum with your name and mailing address.

A description of this clinical trial will be available on http://www.ClinicalTrials.gov; as required by U.S. Law. This Web site will not include information that can identify you. At most, the Web site will include a summary of the results. You can search this Web site at any time.

Future Use of Research Information

In the future, the information that have been collected for this study might be de-identified, which means any information that could be used to identify you will be removed from the information. The de-identified information may be used for future research studies or shared with other researchers. You will not be informed of or asked to consent to these future research activities.

G. WHAT ARE THE POSSIBLE BENEFITS OF TAKING PART IN THIS RESEARCH STUDY?

There is no guarantee that you will receive any benefits from this study. The possible benefit of the exercise program is not known. If you decide to participate in this study, your health will be monitored closely by an exercise trainer who will be working with you through Zoom for each workout session. The knowledge gained from this study may be used to provide information about the benefits of exercise for future BMT survivors.

H. WHAT OTHER OPTIONS ARE THERE?

Your alternative is to not participate in this study. Choosing not to participate will not affect your ability to receive care at City of Hope.

I. ARE THERE ANY PAYMENTS TO YOU FOR TAKING PART IN THE STUDY?

You can receive up to $150 as compensation for your time and effort spent on this study.

You will receive a $50 gift card after completing each assessment on week 0, week 9, and week 17. The $50 gift card on week 17 will not be sent out to you until you have successfully shipped back all the study equipment to us.

J. WHAT ARE THE COSTS?

Neither you nor your insurance carrier will be charged for participation in this study.

K. WHAT HAPPENS IF YOU GET INJURED AS A RESULT OF THIS STUDY?

If you think you have been hurt by taking part in this study, tell the person in charge of this research study as soon as possible. The research doctor's name and phone number are listed in this consent

NOT FOR CLINICIANS USE

form. City of Hope will offer you the care needed to treat injuries directly resulting from taking part in this research. This care will be billed to you or your insurance company. You will be responsible for deductible and co-payments, or any costs not paid by your insurer. There are no plans to pay you or give you other compensation for the injury. You do not give up your legal rights by signing this form.

L. WHAT ARE YOUR RIGHTS IF YOU TAKE PART IN THIS STUDY AND WHAT WILL HAPPEN IF YOU DECIDE NOT TO PARTICIPATE?

Your participation in this research study is voluntary. You are free to withdraw your consent for participation in this study without any loss of benefits, penalty, or interference with any future treatment at City of Hope.

You can decide to stop at any time. Tell a member of the study staff if you are thinking about stopping or decide to stop.

M. CAN YOU BE REMOVED FROM THE STUDY?

You may be removed from this study without your consent for any of the following reasons: you do not follow the investigator's instructions, at the discretion of the investigator, your disease gets worse, or the investigator closes the study. If this happens, the investigator will discuss other options with you.

N. WHOM DO YOU CALL IF YOU HAVE QUESTIONS OR CONCERNS?

The principal investigator, Dr. Kyuwan Lee, is responsible for this study and he is available to answer any questions regarding your participation in this research study. If you have any further questions or in the event of a research related injury, you can contact Dr. Kyuwan Lee at (626) 218-6497.

This study has been reviewed and approved by the Institutional Review Board (IRB). If you have any questions regarding your rights as a research participant, you may contact a representative of that Board, from the Office of Human Research Subjects Protection, at (626) 256-HOPE (4673) ext. 62700.

NOT FOR CLINICIANS USE

O. SIGNATURE SECTION

SIGNATURE FOR CONSENT: By signing this consent form, you are making a decision to participate in this research study. Your signature on this informed consent form indicates that you:

1. Have read and understood the information in this form.
2. Have had the information in this form explained to you.
3. Have had a chance to ask questions and these questions were answered to your satisfaction.
4. Have been informed that you will receive a copy of this signed consent form, which includes the "Experimental Subject's Bill of Rights."

I hereby agree to be a research participant in this research study:

____________________________________ __________ __________

Research Participant's Signature Date Time

(For paper consent only, date and time must be in research participant's handwriting)

____

Print Research Participant's Name

INDIVIDUAL OBTAINING CONSENT SIGNATURE

____________________________________ __________ __________

Signature of Individual Obtaining Consent Date Time

____

Print Name of Individual Obtaining Consent

NOT FOR CLINICIANS USE

Effects of a Telehealth Exercise Intervention to Improve Physical Function and Frailty in Multiple Myeloma Survivors

AUTHORIZATION TO USE AND DISCLOSURE OF YOUR PROTECTED HEALTH INFORMATION (PHI) FOR PURPOSES OF THIS STUDY:

I. **Purpose of this Authorization**: The information about your health is something that is protected by law and cannot, except for certain purposes, be disclosed (shared) without your permission. As part of this research, you are agreeing to allow City of Hope, its affiliated research doctors, healthcare providers, and physician network to use and share with others your protected health information ("PHI"), as needed for the research. If you agree to participate in the study named above (called the "Study"), you must sign this authorization in addition to the *Study Consent Form*.

II. **The Information About You that is Covered By this Authorization**: PHI refers to information that we maintain about you that identifies you and includes the information contained in your medical record. Your medical record consists of information related to your health and the treatment we provide to you, such as your medical history, the results of physical exams, blood tests, x-rays and other diagnostic and medical procedures. If you sign this authorization, you are allowing City of Hope and the individuals indicated below to use and share any PHI we maintain about you that is required for your participation in the Study.

III. **Purposes for Uses and Sharing of your PHI; Who Will Use, Share and Receive your PHI**: Your PHI will be used and shared with others for the purpose of doing this research as described in the *Study Consent Form*. Your PHI will also be used to keep the research sponsor informed about this Study, for reporting to those individuals and authorities responsible for overseeing our research activities to make sure that the activities are properly conducted, and to report to regulatory agencies as required by the Study.

The people authorized to use and share your PHI for purposes of the Study include the Principal Investigator and the research staff supporting the Study; your City of Hope physicians and the health care team; the Health Information Management Services Department (i.e., Medical Records Department), and affiliated research

NOT FOR CLINICAL USE

doctors and other medical centers participating in the research, if applicable. This also includes any agents or contractors used by these individuals or groups for purposes of conducting or managing this Study. At the City of Hope, the Institutional Review Board ("IRB"), and other City of Hope research regulatory committees will have access to your PHI as necessary to monitor research.

You are also allowing your PHI to be shared with the Office for Human Research Protections ("OHRP") and with any person or agency as required by law. In addition, certain other regulatory agencies, including, the Food and Drug Administration ("FDA") will have access to your PHI. Other groups and institutions, including Moterum Technologies, Inc., will also have access to your PHI as necessary for research purposes and to conduct the study.

This authorization will allow us to use and share your PHI for the Study. No other additional uses and disclosures other than for the purposes of the Study is included in this authorization. City of Hope's Notice of Privacy Practices will continue to protect your non-Study information. If necessary, another separate permission will be obtained from you for any non-Study uses or sharing of your PHI.

IV. **<u>Expiration of this Authorization</u>**: This authorization to use and share your PHI will expire twenty-five (25) years from the date that you sign this authorization.

V. **<u>Further Sharing of Your PHI</u>**: Your privacy is important and this is the reason for having rules which control who can use or see your PHI. City of Hope maintains control over your PHI at present, but once we share this information with a third party (for example, an individual or agency outside of the City of Hope), then it is no longer possible to maintain the same level of protection. The persons outside our control may not be governed by federal or state privacy laws and it is possible that they could share your PHI with others for whom you have not given permission.

The information from this Study may be published in scientific journals or presented at scientific meetings but your identity will be kept confidential.

VI. **<u>Your Rights Under this Authorization</u>**: You may cancel this permission to use and share your PHI at any time by contacting City of Hope's Privacy Officer at (626) 256-

NOT FOR CLINICAL USE

HOPE (4673) ext. 64025. You should ask for the form, *Revocation (Cancellation) of Authorization for Use of Protected Health Information for Research*. Fill this form out and return it as the form instructs. Your cancellation begins when the Health Information Management Department of City of Hope receives this form. If you cancel this authorization to use and share your PHI, you will no longer be able to participate in the Study. This is because the research under this Study cannot be conducted without your PHI.

Once you cancel your permission to use and share your PHI, the researchers and others involved in conducting the Study will no longer be able to use or share your PHI for this research. PHI already used and shared up to this point as part of this Study will continue to be used for purposes of this research. This means that any uses of your PHI and any PHI shared about you by City of Hope prior to receiving your cancellation (revocation) form cannot be taken back. While no further PHI about you will be shared for the Study, your PHI already shared will continue to be used in the overall Study.

NOT FOR CLINICIANS USE

VII. **Signing this Authorization is Your Choice:** Your ability to obtain care at the City of Hope will not be affected by your decision to sign this authorization form. You will be able to continue to receive health care at City of Hope if you choose not to sign this authorization form or if you sign this form and later cancel your permission to use and share your PHI.

If you agree to the use and sharing of your PHI, please sign below. You will be given a copy of this authorization form.

NOT FOR CLINICIANS USE

____ __________ __________

Research Participant's Signature   Date   Time

(For paper consent only, date and time must be in research participant's handwriting)

____

Print Research Participant's Name

INDIVIDUAL OBTAINING CONSENT SIGNATURE

____ __________ __________

Signature of Individual Obtaining Consent   Date   Time

____

Print Name of Individual Obtaining Consent
